# Effect of national wealth on BMI: An analysis of 206,266 individuals in 70 low-, middle- and high-income countries

**DOI:** 10.1371/journal.pone.0178928

**Published:** 2017-06-29

**Authors:** Mohd Masood, Daniel D. Reidpath

**Affiliations:** 1 Department of Dentistry and Oral Health, La Trobe Rural Health School, La Trobe University, Bendigo, Australia; 2 Global Public Health, School of Medicine and Health Science, Monash University, Bandar Sunway, Malaysia; Mälardalen University, SWEDEN

## Abstract

**Background:**

This study explores the relationship between BMI and national-wealth and the cross-level interaction effect of national-wealth and individual household-wealth using multilevel analysis.

**Methods:**

Data from the World Health Survey conducted in 2002–2004, across 70 low-, middle- and high-income countries was used. Participants aged 18 years and over were selected using multistage, stratified cluster sampling. BMI was used as outcome variable. The potential determinants of individual-level BMI were participants’ sex, age, marital-status, education, occupation, household-wealth and location(rural/urban) at the individual-level. The country-level factors used were average national income (GNI-PPP) and income inequality (Gini-index). A two-level random-intercepts and fixed-slopes model structure with individuals nested within countries was fitted, treating BMI as a continuous outcome.

**Results:**

The weighted mean BMI and standard-error of the 206,266 people from 70-countries was 23.90 (4.84). All the low-income countries were below the 25.0 mean BMI level and most of the high-income countries were above. All wealthier quintiles of household-wealth had higher scores in BMI than lowest quintile. Each USD10000 increase in GNI-PPP was associated with a 0.4 unit increase in BMI. The Gini-index was not associated with BMI. All these variables explained 28.1% of country-level, 4.9% of individual-level and 7.7% of total variance in BMI. The cross-level interaction effect between GNI-PPP and household-wealth was significant. BMI increased as the GNI-PPP increased in first four quintiles of household-wealth. However, the BMI of the wealthiest people decreased as the GNI-PPP increased.

**Conclusion:**

Both individual-level and country-level factors made an independent contribution to the BMI of the people. Household-wealth and national-income had significant interaction effects.

## Introduction

Obesity has become a significant focus of public health research in virtue of the health sequelae with which it is associated. It was estimated in 2010 that obesity accounted, globally, for 3·4 million deaths, 3·9% of years of life lost, and 3·8% of all disability-adjusted life-years [1]. In general terms, secular changes in population weight are a consequence of long term positive or negative energy balance in individuals, resulting in weight gain and loss, respectively [2]. When individual energy intake from food exceeds individual energy expenditure from physical activity, and this imbalance is maintained over a period, weight is gained; and when the obverse holds, weight is lost. The physiological lens invites an individualistic analysis focusing on biology, behavior, and/or (less commonly) socio-demographics.

In the face of what has been described as the global obesity epidemic, [3, 4] however, an ecological model of individuals within diverse micro, meso, and macro socio-political and economic environments suggests alternative ways of understanding the pathways of population weight gain [5]. The general approach is now well accepted,[6] but can lead to models of enormous complexity [7]. Countries' economic development and economic policy strategies have been investigated as macro-level determinants of increasing levels of obesity [8–10].

A recent study by Neuman and colleagues [8] investigated *inter alia* the relationship between GDP per capita and BMI in 38 low and middle income countries. Findings of the study suggested, on average, BMI increased with increasing national wealth. However, there was a cross level interaction between GDP per capita and individual wealth and BMI. Individuals from the wealthiest quintiles in the poorest countries tended to have an appreciably higher BMI than those in the poorer quintiles, whereas the individuals from the wealthiest quintiles in the very wealthiest of the middle-income countries tended to have a BMI more consistent with the other quintiles of wealth. That is, the BMI of the poorer and the wealthier converged in the wealthiest middle-income countries.

Another study from 40 low- and middle-income countries found that increasing wealth was associated with higher odds of being overweight relative to normal weight [11]. They also found that increasing national wealth was consistently associated with an increased risk of overweight. Unfortunately, the cross-level interaction was not included in the analysis, and we do not know whether the results from the DHS and the WHS would be consistent.

The observation that in low- and middle-income countries increasing national wealth is associated with increasing BMI is consistent with an earlier systematic review [10]. The observed cross-level interaction effect is also consistent with the reviews findings. Unfortunately, there is little research that has looked at countries across the development spectrum from low-income countries through to high-income countries to investigate the relationship between national wealth and BMI.

There is evidence from individual high-income country studies to suggest that obesity rates are higher among poorer and other disadvantaged groups [12, 13]; and this would be consistent with the cross-interaction effect extending into higher income countries. Of the recent studies of national wealth and obesity, only one considered national wealth data from countries across the range of economic development [9]. This study found in a series of unweighted regression analyses of mean, population BMI regressed against GDP per capita, a monotonically increasing relationship between national income and BMI up to a GDP of about USD$30,000 per capita. Unfortunately, there is no way to know how individual wealth within each country affected individual BMI—the issue of the ecological fallacy [14, 15].

While the earlier analysis of WHS data by Nandi and colleagues [11] did not include high-income countries, data from these countries are available. This study explores the relationship between BMI and national wealth and the cross-level interaction effect of national wealth and individual household wealth using multilevel analysis. It was hypothesised that there would be a cross-level interaction effect, and this would extend into the high-income countries.

## Methods

Data were from the World Health Survey (WHS) conducted in 2002–2004, which was launched by the World Health Organization (WHO) to provide nationally representative, valid, reliable and comparable information across 70 low-, middle- and high-income countries from all world regions. WHS is a unique comparable dataset available for 70 countries representing the low-, middle- and high-income countries. In each country, the target population was adults aged 18 years and over, living in private households. Participants were selected using multistage, stratified cluster sampling. This study was approved by the Monash University Human Ethics Committee.

BMI was used as the outcome variable, self-reported height and weight were used to estimate individual level BMI, calculated as weight in kilograms divided by height in meters squared. Several individual and country-level factors were included in the analysis as potential determinants (co-variates) of individual level BMI including, participants’ sex, age, marital-status, education, occupation as well as household wealth and location (rural/urban). Age was measured in years and was centered at mean age of 41.1 years. Marital status was classified as married (including those co-habiting), never married and previously married (separated, divorced and widowed). Education was grouped into three categories; primary school or less, secondary school or college, and higher. Household wealth was determined using a wealth index which classified households based on their ownership of a range of permanent income indicators (household assets). The household items included in the index were the number of rooms in the home, the number of cars, the number of chairs, the number of tables, the presence of electricity; and household ownership of a: bicycle, bucket, washing machine for clothes, washing machine for dishes, refrigerator, fixed line telephone, mobile / cellular telephone, television, computer and clock [16, 17]. A principal components analysis (PCA) was carried out separately for each country to determine the weights to create an index of the asset variables. The weights for the first component were then applied to each person’s data giving a continuous asset index measure [16, 18]. Four break points were created from the PCA score that define wealth quintiles as: Quintile 1(poorest), Quintile 2 (lower-middle), Quintile 3 (middle), Quintile 4 (higher-middle), and Quintile 5 (wealthiest). Occupation was grouped into four categories following the Goldthorpe schema [19]: High (Legislator, Senior Official, Manager, Professional and armed forces), medium (Technician, Associate Professional, Clerk, Service or sales worker), low (Agricultural, fishery worker, Craft, trades worker, Plant/machine operator or assembler) and elementary (elementary workers).

The principal country-level, economic factor used was national income measured by Gross National Income adjusted for purchasing power parity (GNI-PPP) from World Bank data for 2003 [20]. GNI-PPP was centered at mean GNI-PPP at USD 8840. Income inequality was included as a potential confounder. Income inequality was measured using the Gini index based on World Bank data [20]. The Gini index varies from 0 (perfect equality) to 100 (perfect inequality) [21].

Survey analytic procedures were used to account for the complex survey design (stratification and clustering) and incorporate sampling weights to generate population-level estimates and standard errors for each specific country. The R statistical environment (R-3.1.0) was used for the analyses [22]. Lumley's survey package was used for all design-based analyses,[23] and Bates' linear mixed-effect package, lme4 was used for all multilevel, linear regression analyses [24]. The primary analysis was conducted to test the global null hypothesis for all the predictor variables using a two-tailed test and a significance of 0.05. F-values and p-values were calculated for each predictor variable.

A two-level random-intercepts and fixed-slopes model structure with individuals nested within countries was fitted, treating BMI as a continuous outcome. The fixed- and random-parameter estimates for the two-level regression model were calculated using the full maximum likelihood estimation method, as implemented in lme4. Multilevel modeling incorporating survey design features is a matter of ongoing debate with no agreement about the analytic strategy and in any case, unavailable in lme4 [23].

Our modeling strategy was first to estimate the null model (Model 0) and then to include explanatory variables gradually into the model. All individual-level factors were included as explanatory variables in Model 1. Country-level factors (GNI-PPP and Gini index) were included as explanatory variables in subsequent models. The cross level interaction effect between individual level wealth and national income was assessed in final model.

## Results

A sample of 206,266 people from 70 countries was included in this study. Sample size and response rate for each country is given in Table 1. Weighted and unweighted descriptive analysis of individual level variables for is presented in the Table 2. The weighted mean BMI and standard error (SE) in these 70 countries was 23.90 (4.84). Weighted mean age (SE) of the participants was 41.1 (0.17).

**Table 1:** Initial and final sample size after excluding values on height, weight and BMI variables.

Country	Participants surveyed	Participants included in analysis	Response rate
Australia	3600	2915	81.0
Austria	1055	948	89.9
Bangladesh	5552	856	15.4
Belgium	1012	956	94.5
Bosnia and Herzegovina	1028	1022	99.4
Brazil	5000	4443	88.9
Burkina Faso	4825	1725	35.8
Chad	4661	3529	75.7
China	3993	3983	99.7
Comoros	1759	1722	97.9
Congo, Rep.	2497	2193	87.8
Cote d'Ivoire	3184	2854	89.6
Croatia	990	980	99.0
Czech Republic	935	913	97.6
Denmark	1003	974	97.1
Dominican Republic	4534	3111	68.6
Ecuador	4660	4060	87.1
Estonia	1012	998	98.6
Ethiopia	4938	971	19.7
Finland	1013	1004	99.1
France	1008	951	94.3
Georgia	2755	2741	99.5
Germany	1259	1180	93.7
Ghana	3938	3674	93.3
Greece	1000	961	96.1
Guatemala	4770	3193	66.9
Hungary	1419	1399	98.6
India	9994	9268	92.7
Ireland	1014	910	89.7
Israel	1236	1185	95.9
Italy	1000	958	95.8
Kazakhstan	4496	4109	91.4
Kenya	4417	4288	97.1
Lao PDR	4889	4866	99.5
Latvia	856	735	85.9
Luxembourg	700	692	98.9
Malawi	5306	5185	97.7
Malaysia	6040	4989	82.6
Mali	4285	545	12.7
Mauritania	3842	3109	80.9
Mauritius	3888	2509	64.5
Mexico	38746	23480	60.6
Morocco	5000	2041	40.8
Myanmar	5886	5881	99.9
Namibia	4250	3766	88.6
Nepal	8688	3166	36.4
Netherlands	1091	1085	99.5
Norway	984	958	97.4
Pakistan	6379	3449	54.1
Paraguay	5143	4652	90.5
Philippines	10078	8149	80.9
Portugal	1030	896	87.0
Russian Federation	4422	3501	79.2
Senegal	3226	1681	52.1
Slovak Republic	2519	1793	71.2
Slovenia	585	571	97.6
South Africa	2352	1460	62.1
Spain	6364	6161	96.8
Sri Lanka	6732	5663	84.1
Swaziland	3121	1834	58.8
Sweden	1000	975	97.5
Tunisia	5069	4224	83.3
Turkey	11220	8149	72.6
Ukraine	2855	1774	62.1
United Arab Emirates	1180	1132	95.9
United Kingdom	1200	1059	88.3
Uruguay	2991	2965	99.1
Vietnam	3492	3475	99.5
Zambia	3812	2212	58.0
Zimbabwe	4100	2510	61.2
Total	278878	206266	74.0

**Table 2:** Model based and design based descriptive analysis of outcome variable (BMI) and individual level explanatory variables in 70 countries and 53 countries.

	Model Based	Design Based
	n = 206266	N = 885431753
	Mean ± SD	Mean ± SE
Outcome variable		
BMI	24.02(4.84)	23.90(0.07)
Explanatory Variables		
Age	41.19(16.5)	41.11(0.17)
	n(%)	N(%)
Gender		
Female	110778(53.7)	449234978(50.7)
Male	95453(46.3)	436174517(49.2)
Missing values	35(0.016)	22256 (0.1)
Education		
Primary school	101347(49.1)	410420475(46.4)
Secondary school	81964(39.7)	342786029(38.8)
College and above	21894(10.61)	127976371(14.3)
Missing values	1061(0.51)	4248878(0.5)
Marital Status†		
Never Married	40663(19.7)	183696842(20.7)
Married	117864(57.1)	529457230(59.8)
Widowed/Divorced	39129(19.0)	140656180(15.9)
Missing values	8610(4.17)	31621501(3.6)
Household Income		
1^st^ Quintile (Poorest)	40145(19.46)	181004197(20.4)
2^nd^ Quintile	40312(19.54)	175298294(19.8)
3^rd^ Quintile	37709(18.28)	158155749(17.9)
4^th^ Quintile	38032(18.43)	160158090(18.1)
5^th^ Quintile (Wealthiest)	37334(18.09)	142770575(16.1)
Missing values	12734(6.17)	68044846(7.7)
Occupation‡ Ψ		
High	15491(7.5)	67380934(7.6)
Medium	26948(13.1)	119950548(13.5)
Low	53894(26.1)	250461529(28.2)
Elementary	10464(5.1)	46019304(5.2)
Missing values	99469(48.2)	401619438(45.4)
Setting¥		
Urban	105066(50.93)	406861657(46.0)
Rural	94775(46.25)	450418126(50.8)
Missing values	6425(3.11)	28151969(3.2)

^†^All data in this variable was missing for Turkey;

^‡^All data in this variable was missing for Turkey and Norway;

^¥^ All data in this variable was missing for Australia, Netherlands, Norway and Slovenia;

^Ψ^ Occupation categories: High (1. Legislator, Senior Official, or Manager 2. Professional and 10.armed forces), medium (3.Technician or Associate Professional 4. Clerk 5. Service or sales worker), low (6. Agricultural or fishery worker 7. Craft or trades worker 8. Plant/machine operator or assembler) and elementary (elementary workers)

**Fig 1:**
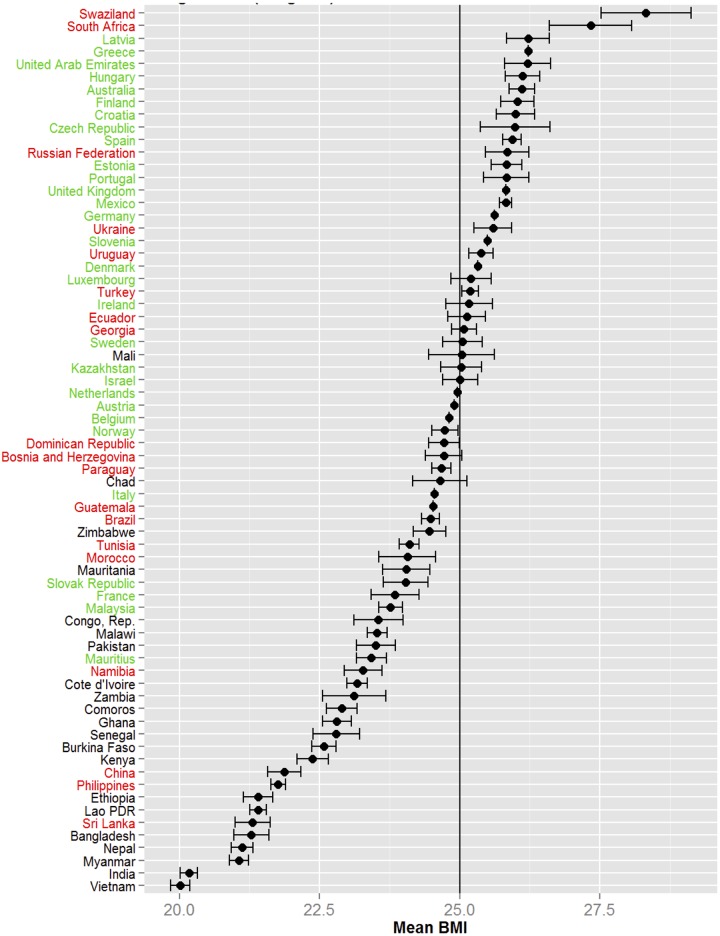
Plot showing the cross-level interaction effect of individual level wealth quintiles and national income.

To analyse the pattern of BMI in across all 70 countries, the design-based mean BMI (with 95% confidence intervals) were calculated (Fig 1). Most low-income countries were at the lower end of the mean BMI, and high- and middle-income countries, at the higher end of the mean BMI. All the low-income countries had a mean BMI below 25.0 and most of the high-income countries had a mean BMI above 25.0. Middle-income countries were scattered in this spectrum from low to high mean BMI.

Model 0, the null model or the variance component model for BMI is shown in Table 3. The fixed part of the model is represented by the coefficient for the constant, which is 24.3 with standard error of 0.20. The random part is given under the heading “Random effect” for variance of level 1 residuals. The estimate of the between-countries variance was 2.75 and the estimate of within-countries variance was 20.07. These estimates for random effect were used to calculate the intra-class correlation coefficient (ICC = 0.125). The ICC suggests that the proportion of total variance that occurs between countries is 0.125. That is to say, 12.5% of the variance in the individual level BMI was between countries and remaining 87.5% of the variation in the individual level BMI was within countries.

**Table 3:** Multilevel multivariate linear regression analysis with individual and country level predictors in 70 countries.

	Model 0		Model 1		Model 2		Model 3	
Fixed Effect	β	95% CI	β	95% CI	β	95% CI	β	95% CI
Intercept	24.3	23.908;24.692	23.5	23.114;23.886	23.4	23.047; 23.753	23.5	23.128;23.872
Country Level								
Log GNI-PPP/10000					0.40		0.48	0.225;0.735
Gini							0.03	-0.009;0.069
Individual Level								
Age			0.034	0.033;0.035	0.034	0.033; 0.035	0.034	0.033;0.035
Gender								
Female			Reference category					
Male			-0.02	-0.059;0.019	-0.02	-0.059; 0.019	-0.02	-0.059;0.019
Education								
Primary school			Reference category					
Secondary school			0.163	0.112;0.214	0.160	0.109; 0.211	0.163	0.112;0.214
College and above			-0.07	-0.150;0.010	-0.07	-0.150; 0.010	-0.07	-0.150;0.010
Marital Status								
Never Married			Reference category					
Married			1.12	1.065;1.175	1.12	1.065; 1.175	1.12	1.065;1.175
Widowed/Divorced			0.70	0.629;0.771	0.70	0.629; 0.771	0.70	0.629;0.771
Household Income								
1st Quintile (Poorest)			Reference category					
2nd Quintile			0.21	0.149;0.271	0.21	0.149; 0.271	0.21	0.149;0.271
3rd Quintile			0.33	0.269;0.391	0.33	0.269; 0.391	0.33	0.269;0.391
4th Quintile			0.41	0.349;0.471	0.41	0.349; 0.471	0.41	0.349;0.471
5th Quintile (Wealthiest)			0.60	0.539;0.661	0.60	0.539; 0.661	0.60	0.539;0.661
OccupationΨ								
High			Reference category					
Medium			-0.075	-0.167;0.017	-0.075	-0.167; 0.017	-0.075	-0.167;0.017
Low			-0.307	-0.397;-0.217	-0.307	-0.397; -0.217	-0.307	-0.397;-0.217
Elementary			0.038	-0.080;0.156	0.039	-0.079; 0.157	0.039	-0.079;0.157
Setting								
Urban			Reference category					
Rural			-0.50	-0.545;-0.455	-0.50	-0.545; -0.455	-0.50	-0.545;-0.455
Random effect	σ	SD	σ	SD	σ	SD	σ	SD
Country level	2.75	1.66	2.3	1.52	1.99	1.41	1.92	1.39
Residual	20.07	4.48	19.38	4.40	19.38	4.40	19.38	4.40
Fit Indices								
AIC	1204429.5		1197180.7		1197172.4		1197172	
BIC	1204460.2		1197385.4		1197387.4		1197397	
Log Likelihood	-602211.8		-598570.4		-598565.2		-598564	
Deviance	1204423.5		1197140.7		1197130.4		1197128	
Model Comparison			With model 0		With model 1		With model 2	
Chi-square (df)	-		7282.8(17)***		10.28(1)**		2.42(1)	
R2			With model 0		With model 0		With model 0	
Country Level R2	-		0.164		0.276		0.302	
Individual level R2	-		0.034		0.034		0.034	
Total R	-		0.050		0.064		0.067	

*pvalue≤0.05;

**pvalue≤0.01;

***pvalue≤0.001

β- regression coefficient; SE- Standard Error; σ- Variance; SD: Standard Deviation; AIC- Akaike information criterion; BIC- Bayesian information criterion; Chisq- Chi Square test; df- Degree of freedom.

^Ψ^ Occupation categories: High (1. Legislator, Senior Official, or Manager 2. Professional and 10. armed forces), medium (3. Technician or Associate Professional 4. Clerk 5. Service or sales worker), low (6. Agricultural or fishery worker 7. Craft or trades worker 8. Plant/machine operator or assembler) and elementary (elementary workers)

The combined effect of all individual level variables on BMI was tested in model 1 (Table 3). Age was positively associated with BMI, every 10 years increase in age was associated with a 0.34 units increase in BMI. Gender was not significantly associated with BMI. On average people with secondary education had higher BMI then people with primary education. Married people had significantly higher BMI than never married and previously married people. Household wealth was also significantly related to BMI. All wealthier quintiles had higher scores in BMI compared with the lowest quintile when, holding all the other variables constant. Professionals and elementary workers did not have significantly different BMIs; however, people in the low occupation category had significantly lower mean BMI than professionals. People living in the rural areas had an average BMI significantly lower than people living in urban areas.

To assess the effect of country level factors on BMI after controlling all individual variables, country level variables including national income and income inequality were added in model 2. First, the association of GNI-PPP with BMI was tested. This showed a 0.4 unit increase in BMI with each USD10,000 increase in GNI-PPP. Later, in model 3 national income and income inequality were added together controlling for all individual level variables. In model 3, the regression coefficient for GNI-PPP remains significant but the regression coefficient for Gini index was not significant. All the individual level variables had similar relationship as in model 2. This model explained 28.1% of country level, 4.9% of individual level and 7.7% of total variance in BMI across the 70 countries.

**Fig 2:**
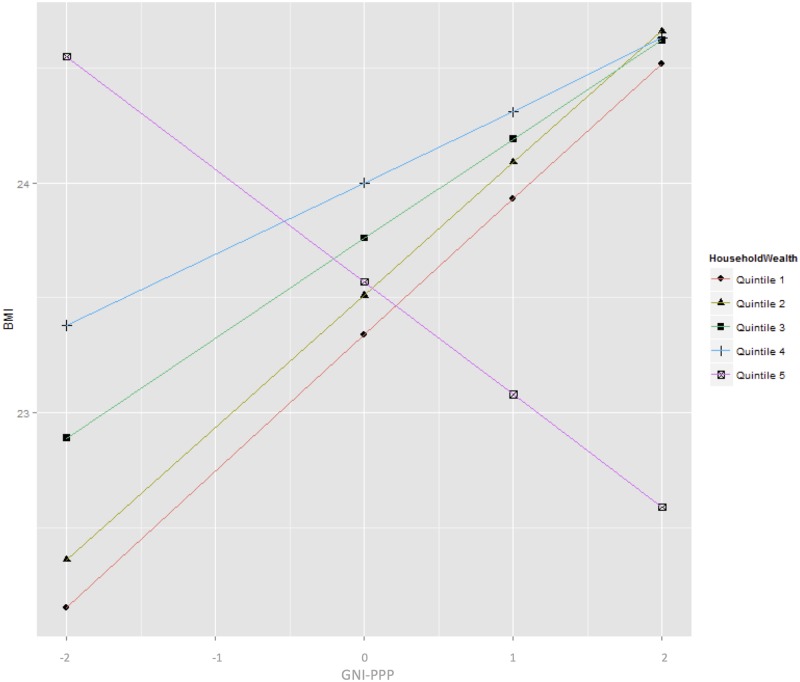
Design based mean BMI (weighted) and confidence interval for the 70 countries.

The cross-level interaction effect between national income and individual level income was modelled to measure the effect of national income on the relationship of individual level income and BMI (Table 4). These results showed a significant interaction effect between all the individual level wealth quintiles and GNIPP except quintile 2. To make results of this model more interpretable I graphically present the interaction effect in Fig 2. This graph shows that as the national income increases people in the first four quintiles show increasing BMI with increasing national wealth. However, the wealthiest quintile shows the reverse pattern. The BMI of the wealthiest people decreases as the national income increases.

**Table 4:** Multilevel multivariate linear regression analysis with individual and country level variables with inter-level interaction between household wealth and national income (GNI-PPP).

	Model 4		Global null hypothesis
Fixed Effect	β	95% CI	F-value (p-value)
Intercept	22.15	20.519; 23.781	
Country Level			
GNI-PPP/10000	0.57	0.315; 0.825	6829(<0.001)
Gini	0.03	-0.009; 0.069	365(<0.001)
Individual Level			
Age	0.034	0.020; 0.048	6451(<0.001)
Gender			93.82(<0.001)
Female	Reference category		
Male	-0.019	-0.062; 0.024	
Education			744.5(<0.001)
Primary school	Reference category		
Secondary school	0.146	0.093; 0.199	
College and above	-0.092	-0.176; -0.008	
Marital Status			1139(<0.001)
Never Married	Reference category		
Married	1.12	1.063; 1.177	
Widowed/Divorced	0.71	0.637; 0.783	
Household Income			93.14(<0.001)
1st Quintile (Poorest)	Reference category		
2nd Quintile	0.21	0.149; 0.271	
3rd Quintile	0.37	0.309; 0.431	
4th Quintile	0.41	0.349; 0.471	
5th Quintile (Wealthiest)	0.60	0.539; 0.661	
OccupationΨ			657.1(<0.001)
High	Reference category		
Middle	-0.067	-0.161; 0.027	
Low	-0.29	-0.382; -0.198	
Elementary	0.052	-0.068; 0.172	
Setting			2670(<0.001)
Urban	Reference category		
Rural	-0.49	-0.535; -0.445	
Household wealth:GNIPPP			1382(<0.001)
1st Quintile (Poorest):GNIPPP	Reference category		
2nd Quintile:GNIPPP	-0.02	-0.079; 0.039	
3rd Quintile:GNIPPP	-0.08	-0.139; -0.021	
4th Quintile:GNIPPP	-0.09	-0.149; -0.031	
5th Quintile (Wealthiest):GNIPPP	-0.26	-0.319; -0.201	
Random effect	σ	SD	
Country	1.93	1.39	
Residual	19.37	4.40	
Fit Indices			
AIC	1197166.6		
BIC	1197432.8		
Log Likelihood	-598557.3		
Deviance	1197114.6		
Model Comparison			
Chi-square(df)	105.77(4)***		
R2			
Country Level R2	0.276		
Individual level R2	0.050		
Total R	0.077		

*pvalue≤0.05;

**pvalue≤0.01;

***pvalue≤0.001;

SE: Standard Error.

β- regression coefficient; SE- Standard Error; σ- Variance; SD: Standard Deviation; AIC- Akaike information criterion; BIC- Bayesian information criterion; Chisq- Chi Square test; df- Degree of freedom.

^Ψ^Occupation categories: High (1. Legislator, Senior Official, or Manager 2. Professional and 10. armed forces), medium (3. Technician or Associate Professional 4. Clerk 5. Service or sales worker), low (6. Agricultural or fishery worker 7. Craft or trades worker 8. Plant/machine operator or assembler) and elementary (elementary workers)

## Discussion

The major finding of this study is that there is a significant cross-level interaction effect between individual wealth and country level wealth and individual BMI. In the four poorest quintiles, increasing individual wealth and increasing national wealth are associated with increasing individual BMI. However, in the wealthiest quintile, BMI steadily decreases with increasing levels of national wealth (Fig 2). This is a potentially important result for several reasons. First, it suggests that earlier results from equivalent studies using exclusively LMIC data, are likely to extend into high-income country data [9]. Second, the results suggest that as countries become wealthier, the BMI outcomes for the bottom 80% of the population become increasingly worse, while the BMI outcomes for the wealthiest 20% of the population become increasingly better. This alone, could have significant consequences for the equity declaration of the Sustainability Development Goals, "to leave no one behind".

The results of this study showed a strong positive association between individual income/wealth and obesity: BMI increased with increases in income/wealth, after adjusting for national income and national income inequality. This global association is similar to the obesity-income/wealth relationship in low- and middle-income countries. In low- and middle-income countries people with higher income/wealth had a higher prevalence of obesity. The majority of the studies, which used income or wealth as an SES indicator showed that wealthier people were more likely to be obese in low- and middle-income countries [10]. An important reason for this trend in 70 WHS countries is that most of the WHS countries were low- and middle-income countries. As most of the countries were low- and middle-income the overall analysis showed a similar pattern to other low- and middle-income country studies.

There are various reasons for the positive association of BMI and wealth in low- and middle-income countries. Household wealth/income enhances the household assets, including owning a car, washing machines, that significantly increased the risk for obesity. Additionally, it has been established that a better economic standing primarily affects obesity in terms of the resources available to buy more food. Therefore, as income increases, households and individuals increase their consumption of food and reduce their energy expenditure, and consequently BMI increases [16, 25].

A shift in income from low to high usually associated with the nutrition transition characterized by a shift towards an unhealthy diet of higher fat and calories and decreased physical activity at work or leisure [17]. In the transition, peoples’ daily diets rely more on animal food sources, and their lifestyles are increasingly sedentary, with less physical activity. Moreover, it could also be linked to excessive consumption of higher calories and fat condensed food (such as animal foods and processed food [17]. In addition, high-income people were at increased risk of snacking and shifting away from traditional healthy cooking patterns to less healthy cooking patterns and less healthy food [18]. Hence, people with higher income and more wealth may increase their risk of obesity.

This study showed a clear gradient in the national income and obesity relationship, where people in poor countries have lower BMI than people in high-income countries. After keeping all other things equal, low- and middle-income countries on average have a lower BMI than high-income countries. Every 10,000 USD increase in GNI-PPP is associated with 0.3 unit increase in BMI. These results are in agreement with previous cross-national studies identifying a positive association between obesity and national income [9, 26–28]. A positive correlation between national income and BMI exists, with the prevalence of obesity being greater in developed countries than less developed countries, and obesity rates increasing as per capita incomes increases [29, 30]. However, some previous studies showed no association of BMI and national income, but the majority of these studies were based only on high-income countries [31].

The positive associations between high national income and higher BMI or obesity are attributed to differences in lifestyle behaviours that accompany economic development and urbanization (e.g., alterations in the quantity and sources of caloric intake, and changes in physical activity). While its main proximate cause has been identified as a surge in extra-meal snacking and secondary eating consumption (including eating more, and buying more entertainment and energy saving devices), a decline in physically demanding labour [32], changes in food production technologies and prices have all been found to contribute to obesity development [33, 34].

While income inequality was treated as a potential confounding variable in this study, and was not the specific focus of any hypothesis, there has been sufficient research looking at income inequality as a putative cause of health inequality that the results are worthy of consideration here [26, 27, 31, 35, 36]. After controlling for national income, household income, and other individual level factors, national income inequality was not significantly associated with BMI. It is possible that the effects of the income inequality are already subsumed by income/wealth at the individual-level and national income at the country-level [16]. It is also possible that the country level income inequality is simply not associated with BMI. It is perhaps the absolute income of a person and the absolute income of a country that makes unhealthy/health food accessible or unhealthy/health lifestyle accessible.

These results are in contrast with the majority of the earlier literature on income inequality and health [31, 33]. The positive correlation between income inequality and obesity prevalence was observed in most developed countries including the U.S. [13], Europe [26], and OECD countries [31]. Many studies by Wilkinson and colleagues reported the detrimental effect of income inequality on health (mortality, morbidity and self-reported health status) in the OECD countries [37]. As this evidence was predominantly from high-income countries, it is possible that the positive association between income inequality and poor health reported by Wilkinson and colleagues only have effect in the high-income countries where Gini is low, but not for the low- and middle-income countries. However, the inverse Gini effect on obesity has also been observed for some developing countries such as China and India [38]. On the other hand, there are studies that found no significant relationship between income inequality and health [27, 35, 39, 40].

Most of the countries included in this study had good response rates of more than 60%, with the exception of Bangladesh and Ethiopia. Achieving high response rates in national surveys is always challenging, especially for low- and middle-income countries. Lack of information on non-respondents and exclusion of these non-respondents for weight or height is a limitation of this study. However, the extent of the bias, if any, which could have been introduced could not be assessed.

### Conclusion

Both individual-level and country-level socioeconomic factors make an independent contribution to the BMI of the population. In the view of income inequality, household income and national income have independent, albeit unequal effects on obesity. The pattern is consistent, regardless of the other individual level factors. Meanwhile, the association between income inequality and obesity risk warrants further investigation.

**Key Points**

- The major finding of this study is that there is a cross-level interaction between individual wealth and country level wealth and individual BMI.
- As countries become wealthier, the BMI outcomes for the bottom 80% of the population become increasingly worse.
- While the BMI outcomes for the wealthiest 20% of the population become increasingly better.
